# Femoral-Facial Syndrome

**Published:** 2014-08-06

**Authors:** Monique-Terese Squiers, Molly Rideout, Donald Laub

**Affiliations:** University of Vermont College of Medicine, Burlington, Vt

**Keywords:** cleft, diabetic, femoral, facial, syndrome

## DESCRIPTION

**Figure 1:**
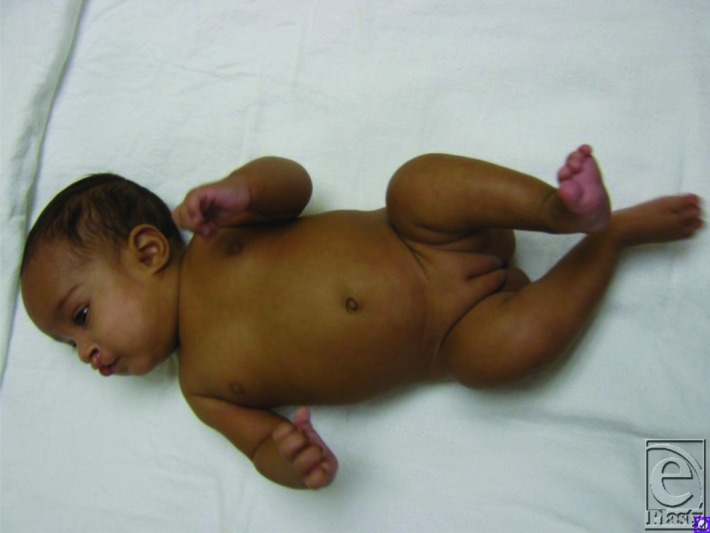
D.H. at 3 months of age, demonstrating bilateral shortened and bowed femurs.

**Figure 2:**
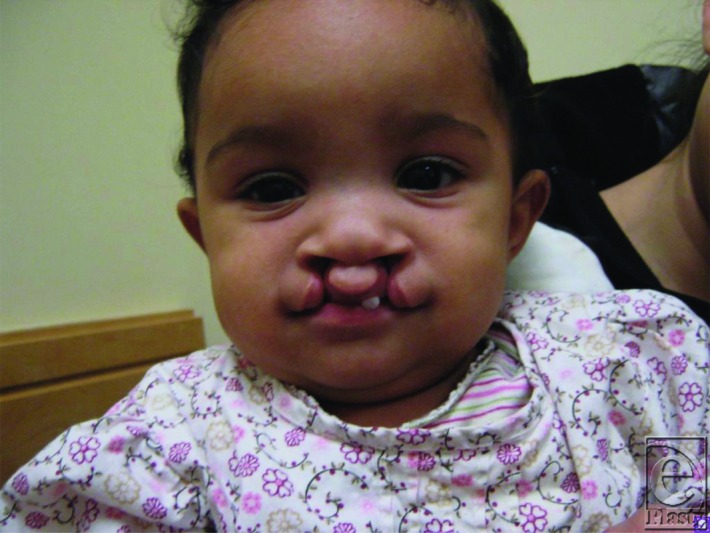
D.H. at 3 months of age, demonstrating bilateral cleft lip with cleft alveolus.

D.H. is a female infant diagnosed by antenatal ultrasound with bilateral bowed and shortened of femurs (Fig 1) and bilateral cleft lip (Fig 2). She was born preterm to a 31-year-old African-American woman with poorly controlled diabetes mellitus. After birth, she was diagnosed with tethered cord and mild ventriculomegaly.

## QUESTIONS

1. **What anomalies are associated with femoral-facial syndrome (FFS)?**
2. **What is the etiology of FFS?**
3. **How early in gestation may FFS be detected?**
4. **What is the association between FFS and maternal diabetes?**

## DISCUSSION

**Figure 3:**
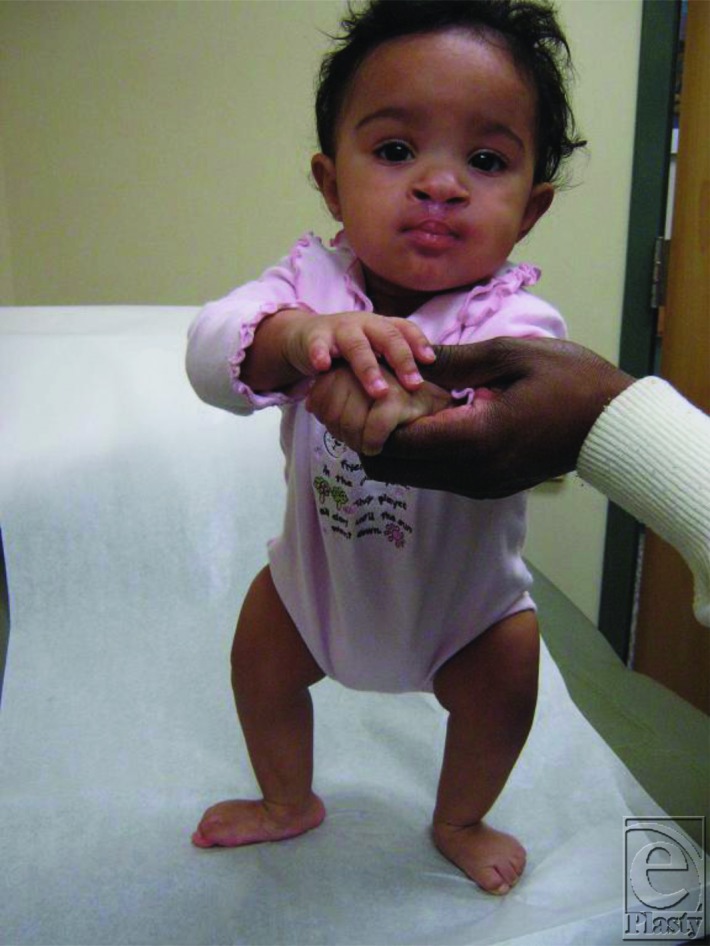
D.H. at 9 months of age, after cleft lip repair.

Daentl et al first reported 6 patients with a constellation of malformations including bilateral hypoplastic femurs, up-slanting palpebral fissures, short nose, long philtrum, thin upper lip, micrognathia, and cleft palate in 1975.^1^ He called the syndrome femoral hypoplasia/unusual facies syndrome, but it is currently termed *FFS*. At least 62 cases of FFS have been documented since 1975, and only a few of these have had congenital central nervous system malformations.^2^^,^^3^ Children with FFS have short stature due to shortened femurs (Fig 3). Radiographic abnormalities are usually asymmetric. Patients frequently have hypoplastic fibulae and acetabulae, as well as hypoplastic or aplastic femurs.^4^ Vertebral abnormalities are seen in up to 35% of patients with FFS and may include scoliosis, hemivertebrae, synostosis, spina bifida occulta, or malsegmentation of the sacrum. Genitourinary anomalies have been reported in up to 60% of patients with FFS.^4^

The etiology of FFS is unknown. Although there have been reports of autosomal dominant inheritance,^5^ multifactorial inheritance is actually much more likely,^6^ and most cases appear sporadically.

Fetal ultrasound can detect femoral growth arrest as early as 13 weeks of gestation.^7^ D.H.'s antenatal imaging at 19 weeks' gestation revealed oligohydramnios, intrauterine growth restriction, bowed and angulated femurs with malpositioned femoral heads suggestive of Caudal Regression Syndrome, bilateral cleft lip/palate, and left ventriculomegaly. Thus, a prenatal diagnosis of FFS was made.

An association with maternal diabetes mellitus is seen in approximately 35% of cases.^4^^,^^8^ The syndrome is analogous to Caudal Regression Syndrome, which may also be associated with cleft lip and palate, and also has a strong association with maternal diabetes mellitus.^4^

Our patient, D.H., was noted to have a sacral dimple, and magnetic resonance imaging confirmed the presence of a tethered cord. She had a surgical repair of her cleft lip and a surgical release of her tethered cord, both without complications. Her hip dysplasia has been managed nonoperatively. This case demonstrates one of a variety of presentations of FFS with femoral bowing and shortening and complete bilateral cleft lip and is additionally notable for congenital CNS malformations. It represents another case associated with gestational diabetes but presents without ocular involvement.
